# P-1413. Targeted Next-Generation Sequencing for Pre-XDR/XDR-TB Diagnosis: A Cost-Effectiveness Analysis in South Africa, India, and Georgia

**DOI:** 10.1093/ofid/ofaf695.1600

**Published:** 2026-01-11

**Authors:** Ginenus Fekadu, Tadesse Tolossa, Tesfaye Regassa Feyissa, Sze Chai Chan, Nathorn Chaiyakunapruk, Shanquan Chen, Martin Siegel, Wai-Kit Ming

**Affiliations:** Wollega University, Nekemte, Oromiya, Ethiopia; Deakin University, Geelong, Victoria, Australia; Deakin University, Geelong, Victoria, Australia; City University of Hong Kong, Hong Kong, Not Applicable, Hong Kong; University of Utah, Salt Lake City, Utah; London School of Hygiene & Tropical Medicine, London, England, United Kingdom; Technische Universität Berlin, Berlin, Berlin, Germany; City University of Hong Kong, Hong Kong, Not Applicable, Hong Kong

## Abstract

**Background:**

Drug-resistant tuberculosis (DR-TB), particularly pre-extensively and extensively drug-resistant TB (pre-XDR/XDR-TB), is a critical global health challenge. Phenotypic drug susceptibility testing (pDST), the gold standard, has prolonged turnaround times, while targeted next-generation sequencing (tNGS) rapidly identifies resistance-conferring mutations. This study evaluates the cost-effectiveness of tNGS-based strategies for diagnosing pre-XDR/XDR-TB among rifampicin-resistant TB (RR-TB) patients in South Africa, India, and Georgia.

**Methods:**

A decision-analytic model—combining a short-term decision tree and a 10-year Markov component —simulated outcomes for RR-TB cohorts under three strategies: (a) pDST, (b) tNGS, and (c) a combination of pDST and tNGS. Outcomes included direct medical costs, quality-adjusted life-years (QALYs), early treatment rates, mortality, and incremental cost-effectiveness ratios (ICERs). Inputs were derived from public databases and published literature. Cost-effectiveness was evaluated against country-specific GDP-based willingness-to-pay (WTP) thresholds, with sensitivity analyses assessing the uncertainty.

**Results:**

tNGS-based strategies improved early treatment initiation and reduced mortality across all settings. In South Africa, tNGS was cost-effective (ICER: $2,805/QALY; WTP: $6,253/QALY). In India, neither tNGS ($4,453/QALY) nor the combination ($6,198/QALY) met cost-effectiveness (WTP: $2,485/QALY). In Georgia, the combination strategy was preferred (ICER: $6,361/QALY; WTP: $8,120/QALY). Key cost-effectiveness drivers included the hazard ratio for mortality from delayed treatment and tNGS costs. Probabilistic sensitivity analysis (10,000 Monte Carlo simulations) showed tNGS had 58% cost-effectiveness probability in South Africa, whereas pDST (50%) and combination strategies (42%) were preferred in India and Georgia, respectively.

**Conclusion:**

tNGS is cost-effective for pre-XDR/XDR-TB diagnosis in South Africa and Georgia but not in India under current economic conditions. The findings underscore context-specific benefits, including earlier treatment and reduced mortality. Policymakers should balance upfront costs with long-term gains, prioritizing local healthcare needs.

**Disclosures:**

All Authors: No reported disclosures

